# Structural Changes Induced by Pulsed Electric Fields Increase the Concentration of Volatiles Released in Red Onion (*Allium cepa* L. var. Red Pearl) Bulbs

**DOI:** 10.3390/foods8090368

**Published:** 2019-08-26

**Authors:** Thirawat Tantamacharik, Sze Ying Leong, Michelle J. Leus, Graham T. Eyres, David J. Burritt, Indrawati Oey

**Affiliations:** 1 Department of Food Science, University of Otago, PO Box 56, Dunedin 9054, New Zealand; 2 Riddet Institute, Private Bag 11 222, Palmerston North 4442, New Zealand; 3 Department of Botany, University of Otago, PO Box 56, Dunedin 9054, New Zealand

**Keywords:** onion microstructure, pulsed electric fields, cell viability, volatiles, dipropyl disulfide

## Abstract

This study investigated whether pulsed electric field (PEF) treatment can induce structural changes of whole, intact red onion bulb (*Allium cepa* L. var. Red Pearl). Onion bulbs were treated at electric field strengths of 0.6 and 1.2 kV/cm combined with energy inputs of 6 and 60 kJ/kg at different onion orientations with respect to the high voltage electrode. Results showed that onion cells across all fleshy scales experienced uniform cell damage with a higher proportion (>80%) of non-metabolically viable cells after PEF treatment at 1.2 kV/cm when the root end was positioned facing toward the PEF electrode. The findings were supported by cryogenic-scanning electron micrographs (cryo-SEM), where the underlying storage circular cells were completely damaged owing to the PEF treatment. In this study, it was found that the treatment intensity of PEF to induce structural damage across all the scale layers of an onion bulb coincided with an increase in dipropyl disulfide (DPDS) released from the onion bulbs. Therefore, DPDS was used as a volatile marker indicating cellular disruption within whole, intact onion bulbs. A considerable increase of DPDS, up to 52-fold, was detected from PEF-treated onion bulbs compared to untreated bulbs.

## 1. Introduction

Onion (*Allium cepa* L.) is one of the most important horticultural vegetables widely used in daily cooking as a versatile flavor ingredient. Onion bulbs, either from red, purple, white, brown, or yellow cultivars, are a good source of vitamin C and health-promoting phenolic compounds such as flavonoids [1]. Alk(en)yl cysteine sulfoxides (ACSOs), which are sulfur-containing compounds in the form of cysteine derivatives, are the next most abundant bioactive constituents found in onions that have perceived biological activities, i.e., anticarcinogenic, antimutagenic, antimicrobial, and antioxidant effects in promoting human health [2]. ACSOs are more commonly documented as the important flavor precursors in onions where they act as substrates for enzyme alliinase/alliin lyase (EC 4.4.1.4) [3]. Enzymatic hydrolysis of ACSOs, as catalyzed by alliinase, takes place immediately upon tissue disruption (e.g., produced by peeling, cutting, cooking, or mastication) and generates products comprised of sulfenic acid, pyruvate, and ammonia [4]. Following this, sulfenic acids undergo spontaneous reactions amongst themselves and with other compounds resulting in the formation of a complex mixture of sulfur-containing volatile compounds, i.e., thiosulfinate, thiosulfonates, thiols, and mono-, di-, tri-, and poly-sulfides, which provide onions with their unique odor and flavor characteristics [3]. 

The processing of onions, under certain conditions, may facilitate alliinase-catalyzed reactions, which increase the generation of key onion volatiles and produce the range of end products to enhance the onion flavor. In previous work, gamma-irradiation of dried onion powder at a dose level of 10 kGy has been shown to increase the contents of dimethyl disulfide, methyl propyl disulfide, dimethyl trisulfide, and methyl propyl trisulfide [5]. Another recent study has demonstrated that high pressure processing (200 MPa at 25 °C for 5 min) of diced brown onions was able to preserve the total content of the volatile concentration and even increase the concentrations of dipropyl disulfide (DPDS), methyl propyl trisulfide, and dipropyl trisulfide [6]. Nonetheless, it should be noted that increases in the concentration of the majority of sulfur-containing volatiles could be because the onion tissues have already experienced considerable tissue damage (e.g., pulverizing and dicing step) prior to irradiation or high-pressure processing. Therefore, these results may not be conclusive to deduce whether the formation of sulfur volatiles is predominantly due to tissue disruption induced by the processing technique. 

Pulsed electric fields (PEF) processing technology has the potential to produce cell membrane breakdown, leading to changes in the membrane permeability of plant materials [7], loss of cell integrity, and absence of cell compartmentalization [8]. Consequently, this can promote an interaction between enzymes and substrates that allows various biochemical reactions to occur. Therefore, PEF-induced cell damage across all onion cells within the bulb is expected to trigger numerous enzymatic reactions. An electric field strength of 0.33 kV/cm and above is reported to be adequate to rupture most of the onion cells and cause irreversible cell damage [9,10,11]. Recent studies have consistently shown that PEF is capable of causing structural damage to intact/whole solid plant materials made up of highly complex and heterogeneous tissues such as potato tubers [8], sweet potato tubers [12], oca tubers [13], and bunching onions [14]. However, structural- and quality-related changes on a whole onion bulb under the influence of PEF is not understood.

The main objective of this study was to investigate how structural changes induced by PEF treatment on intact and whole red onion bulbs (*Allium cepa* L. var. Red Pearl) could trigger the alliinase associated reactions, leading to an increased production of onion volatiles. Whole onion bulbs were treated with PEF at varying intensities of specific energy input (6 vs. 60 kJ/kg) and electric field strengths (0.6 vs. 1.2 kV/cm) with different onion orientations (root end of the onion bulb facing vs. not facing the high voltage electrode). Cryogenic scanning electron microscopy (cryo-SEM) was used to visualize PEF-induced cell/tissue damage. Increases in the concentration of onion volatiles were evaluated as a function of time following PEF treatment (6 h) using headspace solid-phase microextraction gas chromatography–mass spectrometry (SPME-GC-MS). With the aid of kinetic modelling, the related kinetic parameters indicating the rate of volatile changes in response to PEF treatment were estimated. 

## 2. Materials and Methods

### 2.1. Onion Bulbs

**Figure 1:**
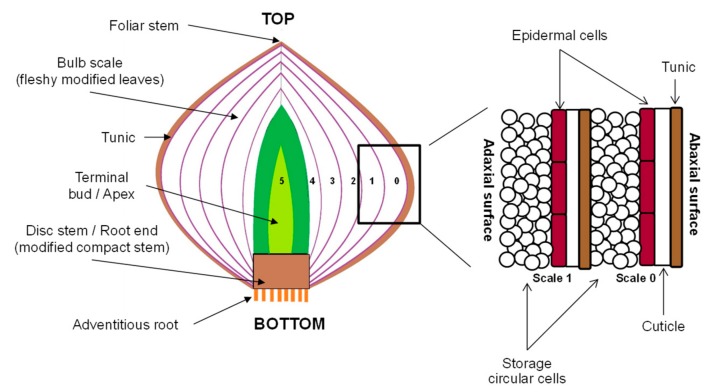
Schematic structure of an onion bulb.

Commercial red onions (*Allium cepa* L. var. Red Pearl) were provided by a local grower (Lincoln, New Zealand). The onion bulbs were screened for similarity in appearance (uniform distribution of purplish-red pigments on the outermost fleshy scale), weight (average of 48.57 ± 8.31 g after removal of the outer tunic and the outermost scale (scale 0, Figure 1)), and diameter and height (less than 50 mm). Damaged, fungal-infected and sprouting onion bulbs were excluded from this study. Only onions with acceptable quality were stored at 10 ± 2 °C in the dark and well-ventilated conditions, and were kept for no longer than 14 days before PEF treatments.

### 2.2. Pulsed Electric Field Treatments

**Figure 2:**
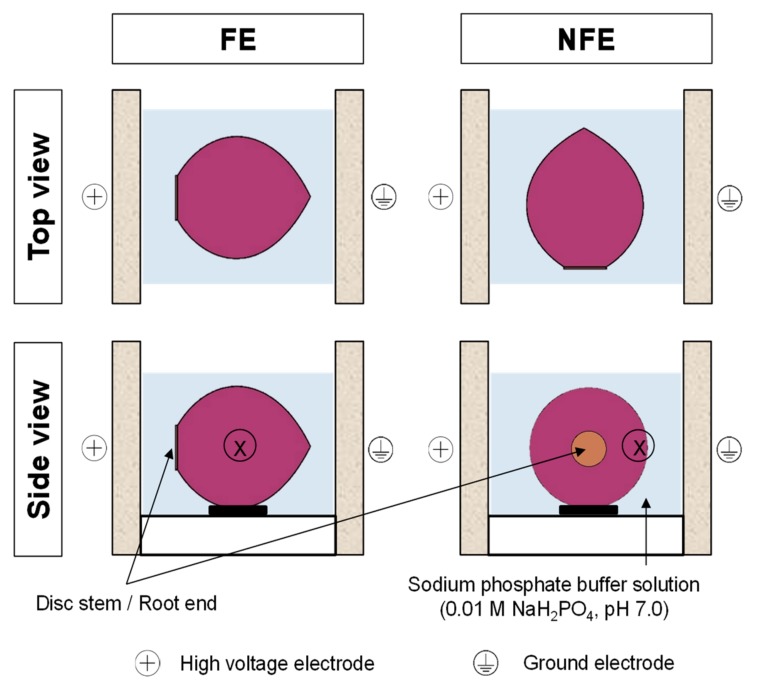
Orientation of an onion bulb positioned inside the PEF chamber during treatment. The onion bulb was positioned with either the root end facing the electrode (FE) or the root end not facing the electrodes (NFE). “X” indicates the equatorial area of the onion bulb where specimens were later taken for cryogenic-scanning electron micrographs (cryo-SEM) evaluation.

Before PEF treatment, the outer “papery” scales (also known as tunic layers, Figure 1) and the outermost fleshy scale (scale 0, Figure 1) of the onions were carefully removed by hand and the bulbs were washed with water. For every PEF treatment, an onion bulb (48.57 ± 8.31 g) was positioned in the middle of the PEF treatment chamber (Figure 2). The PEF chamber (German Institute of Food Technologies, Quakenbrück, Germany) consisted of two parallel stainless-steel electrodes (100 mm length × 50 mm height × 5 mm thick each) with an 80 mm gap between them (electrode gap). The onion was fully immersed in a sodium phosphate buffer solution (0.01 M NaH_2_PO_4_, pH 7.0, with an electrical conductivity of 1.4 mS/cm), which always resulted in an onion-to-buffer ratio of 50:250 in the treatment chamber. The onion was then immediately treated using the PEF apparatus (ELCRACK HVP 5 system; DIL, German Institute of Food Technologies, Quakenbrück, Germany) under a batch treatment configuration. 

In the present study, onions positioned at two different orientations with respect to the PEF electrodes were compared (Figure 2), in which an onion bulb was positioned with either the root end (or disc stem) facing the high voltage electrode (hereafter referred to as “FE”) or the root end not facing the electrodes (hereafter referred to as “NFE”). Untreated onion bulbs were used as controls (hereafter referred to as “No PEF” onions), and similar to PEF-treated onions, they were submerged in the sodium phosphate buffer at the same onion-to-buffer ratio for no more than 3 min until further use.

For each onion orientation, onions were treated independently with three different PEF conditions (considering the intensity of the electric field strength applied and pulse number required to achieve the required specific energy input) and compared to untreated (“No PEF”) samples as a control (Table 1). The PEF 1 treatment involved treating onions with an electric field strength of 0.6 kV/cm in conjunction with a specific energy input of 6 kJ/kg. For the PEF 2 treatment, onions were treated at the same level of field strength (0.6 kV/cm), but the specific energy input was increased 10-fold to 60 kJ/kg. Onions were also treated at a higher field strength of 1.2 kV/cm and a specific energy input of 6 kJ/kg (PEF 3). These PEF conditions fall within the range of electric field strengths and specific energy inputs that have been shown capable of rupturing the plasma membrane of onion cells [15] and substantially impacted on the cell integrity of the onion [14,16]. Input voltages and the number of square wave bipolar pulses were adjusted to achieve the aforementioned treatment intensities, while a constant pulse width of 20 μs and a frequency of 50 Hz were used for all the PEF processing conditions applied. Experiments were carried out at 20 °C and temperature changes due to PEF treatments were found to be negligible. For every PEF processing condition tested in this study, at least three independent PEF experiments using individual whole onion bulbs were conducted (*n* = 3).

Untreated and PEF-treated onions, along with the sodium phosphate buffer, were transferred to an enclosed container (LabServ, Thermo Fisher Scientific, Scoresby, VIC, Australia) where onions were removed for photo capturing (as detailed below) after 0, 1, 2, 4, and 6 h of steeping in the sodium phosphate buffer. Moreover, a small volume of buffer (5 mL) was removed simultaneously at each steeping time point for headspace volatile analysis (see Section 2.5). At each steeping time point, onion bulbs were wiped clean to remove surface water and their appearance was captured using a Canon PowerShot SX50 HS digital camera (Tokyo, Japan) on a copy stand unit (Durst Phototechnik AG, Brixen, Italy) at the same position and under constant light conditions. The captured images were visually compared for changes in distribution of the purplish-red pigments at the outermost fleshy scale of the bulbs over the course of 6 h of steeping after the PEF treatment. 

### 2.3. Microscopic Evaluation Using Cryo-Scanning Electron Microscopy (Cryo-SEM)

Two tissue specimens (6 mm diameter) were obtained from the equatorial area (Figure 2) of the second and third fleshly scales (scales 2 and 3, Figure 1) of the same onion bulb, either untreated or PEF-treated, using a cork borer. One specimen was evaluated at the abaxial (outer waxy, concave shape) surface of the onion epidermis, and the other specimen was observed at the adaxial (inner, convex shape) surface, with the tunic layer removed. The specimens were rinsed using a sodium phosphate buffer (0.01 M NaH_2_PO_4_, pH 7.0) to remove surface cytosol and to prevent the remnant cytosol from forming a crystal sheet on the specimen surface during sample preparation and freezing with liquid nitrogen that could interfere with the subsequent surface morphology imaging. Each specimen was glued onto an aluminum disc using a Tissue-Tek optimum cutting temperature compound (Sakura Finetek Inc., Torrance, CA, USA), plunged into liquid nitrogen, and stored in liquid nitrogen until viewing. The specimen was transferred to a Gatan Alto 2500 cryo-preparation chamber (Gatan Inc., Pleasanton, CA, USA) using a brass cryo-holder. The specimen was left to stand in the chamber to allow ice crystals on the surface of the specimen to sublime at −85 °C under a vacuum condition. After that, the surface morphology of the specimen was viewed and captured in a JOEL JSM-6700F field emission scanning electron microscope (JOEL Ltd., Tokyo, Japan) at 100× magnification and a low accelerating voltage of 3 or 5 kV.

### 2.4. Determination of Cell Viability Using Tetrazolium Salt as a Staining Agent

A 2,3,5-triphenyltetrazolium chloride solution (0.5% *w*/*v*) was freshly prepared and protected from light exposure to avoid precipitation and a pH change. Onion bulbs, either untreated or PEF-treated, suspended in the sodium phosphate buffer for a total contact time of 6 h were used for the staining analysis. This was because these onions had reduced natural purplish-red pigments distributed on the abaxial (outer waxy) surface of fleshy scales before staining. Therefore, the subsequent image analysis was not interfered by the presence of natural pigments in the scales. Each onion bulb was cut in half from the vertical axis of foliar stem and the fleshy scales were gently detached by hand. Four layers of fleshy scales (scale 1–4) and a terminal bud were obtained from each onion bulb. Individual onion scales were each completely submerged in 100 mL tetrazolium solution on a 90 × 25 mm petri dish (LabServ, Thermo Fisher Scientific, Scoresby VIC, Australia) for 24 h in the dark to allow for complete diffusion of the tetrazolium salt into the onion cells. Reduction of tetrazolium salt using oxidoreductase enzymes, present in living cell mitochondria, into the insoluble red formazan is directly proportional to the number of metabolically active viable cells [17]. Therefore, cells with formazan deposits are considered as viable cells, but this is not the case for non-viable/dead cells.

Prior to photo capturing, the edges of the stained onion scales were cut to flatten the convex scales and maximize the total surface area for image analysis. The photos of stained onion fleshy scales, with the adaxial (inner/convex) surface facing upwards, were captured in the same manner as mentioned previously (Section 2.2). The stained area (in cm^2^) representing the viable cells was measured using an image processing software (ImageJ 1.48v software package; National Institutes of Health, Bethesda, MD, USA), where the color threshold function was employed to estimate the difference in the degree of cell viability between untreated and PEF-treated samples. Since the color of natural purplish-red pigments of onion and the red formazan deposits due to staining were rather similar, values of hue (0–255), saturation (125–255), and brightness (1–255) were set using the color threshold function to a range that the software could no longer detect the natural purplish-red pigments present in the onion scale.

### 2.5. Determination of Onion Volatile Compounds Using Headspace Solid-Phase Microextraction Coupled with Gas Chromatography–Mass Spectrometry (HS-SPME-GC-MS)

In the present study, the sodium phosphate buffer (0.01 M NaH_2_PO_4_, pH 7.0) that the onion bulb was suspended in was used as the medium to measure onion volatile compounds generated and released from the samples. This method was applied to understand the impact of PEF treatment on volatile compounds generated as a result of tissue disruption in whole, intact onion bulbs without any confounding impact of further disruption during sample preparation. Samples were taken immediately (0 h), then 1, 2, 4, and 6 h after PEF treatment from the same onion bulb. At each sampling point, 5 mL of phosphate buffer was transferred into a 20 mL headspace vial containing 1.5 g sodium chloride (BDH Chemicals, Poole, England), and the vial was immediately sealed with a PTFE-coated silicon septum screw cap. The headspace vial was heated at 40 °C and agitated for 5 min (PAL3 RSI 85, CTC Analytics AG, Zwingen, Switzerland) in order to aid the partitioning of volatile compounds into the headspace. Volatile compounds were then extracted using solid-phase micro-extraction (SPME) with a divinylbenzene/carboxen/polydimethylsiloxane (DVB/CAR/PDMS) fiber (Supelco, Bellefonte, PA, USA) for 20 min at 40 °C.

Volatile compounds were analyzed using gas chromatography–mass spectrometry (GC-MS) with an Agilent 6890N GC and 5975B VL MSD (Agilent Technologies, Santa Clara, CA, USA). The extracted compounds on the SPME fiber were desorbed for 5 min in the injection port at 230 °C under split-less mode (2 min), then with a purge flow to a split vent (50 mL/min). Separations were achieved on a Zebron ZB-Wax column (60 m × 0.32 mm I.D. × 0.5 µm d.f.; Phenomenex, Torrance, CA, USA) using a helium carrier gas at a 1.2 mL/min constant flow. The initial oven temperature was 50 °C, then ramped to 100 °C at 5 °C/min, then to 240 °C at 10 °C/min, and maintained at 240 °C for 5 min. The mass spectrometer was operated with a mass range of 30–300 *m*/*z*, with the source temperature at 230 °C and MS quadrupole temperature at 150 °C. The National Institute Standards and Technology (NIST) 2014 mass spectra database was used to identify the volatile compounds, supported by comparison with earlier publications [6,18]. The extracted ion chromatogram peak area of each identified compound was manually integrated using specific mass ions and quantified for comparison according to Nandakumar et al. [14].

### 2.6. Kinetic Modelling and Parameter Estimation for Volatiles Release of PEF-Treated Onion Bulbs

The number of volatiles detected in the headspace of the phosphate buffer steeped with either untreated or PEF-treated onions was determined as a function of the steeping duration (from 0 to 6 h). The general equation of an *n*^th^ order rate of reaction (*r*) expressed as Equation (1) [19] was used to estimate the order of the reaction, where *A* is the concentration (based on the peak area) of the volatile compound at time *t* and *k* is the rate constant:

(1) $$\mathrm{Rate}\;\mathrm{of}\;\mathrm{reaction}\;(r)=-\frac{dA}{dt}={kA}^{n}$$

In the present work, a zero-order reaction was used for kinetic modelling of the changes in volatile concentration detected at the headspace of the phosphate buffer during steeping with untreated or PEF-treated onions. Integration of Equation (1) with respect to time for *n* = 0 (for zero-order kinetics) leads to Equation (2), where *A*_0_ indicates the initial concentration of the volatile compound at time, *t* = 0 h:

*A* = *A*_0_ − *kt* (2)

The selection of zero-order kinetics used in this study was based on visual inspection of different concentration plots as a function of time. Moreover, the suitability of a zero-order kinetic model was evaluated by visual inspection of fitting and the random distribution of residuals between a measured data point and the predicted values generated from the linear regression analysis (SAS Statistical Software version 9.4, SAS Institute Inc., Cary, NC, USA). Another criteria to consider for the data fitting into a zero-order kinetic model is based on the calculation of the coefficient of determination (corrected R^2^, Equation (3)) [20].

(3) $${\mathrm{Corrected}\;R}^{2}=1-\frac{\left[\left(m-1\right)\;\left(1-\frac{{\mathrm{SSQ}}_{\mathrm{Reg}}}{{\mathrm{SSQ}}_{\mathrm{Total}}}\right)\right]}{\left(m-j\right)}$$

where *m* is sum of observations (or degree of freedom for total observations), SSQ_Reg_ is the sum of the squares of the model, SSQ_Total_ is the sum of the squares of total observations, and *j* is the sum of the parameters. The corresponding kinetic parameters and asymptotic standard error of the estimated parameter at a 95% confidence interval were estimated based on linear regression analysis (SAS version 9.4) using the integrated volatile dataset from the three independently treated onions.

### 2.7. Data Analysis

Results are presented as mean ± standard error for three independent measurements. An analysis of variance (ANOVA) was applied using SPSS version 22 (IBM Corporation, New York, NY, USA) followed by a Tukey honestly significant difference (HSD) post-hoc test to compare the means (*p* < 0.05).

## 3. Results

### 3.1. Effect of PEF on the Changes in the Purplish-Red Pigments Distribution at the Exterior of Onion Bulbs

**Figure 3:**
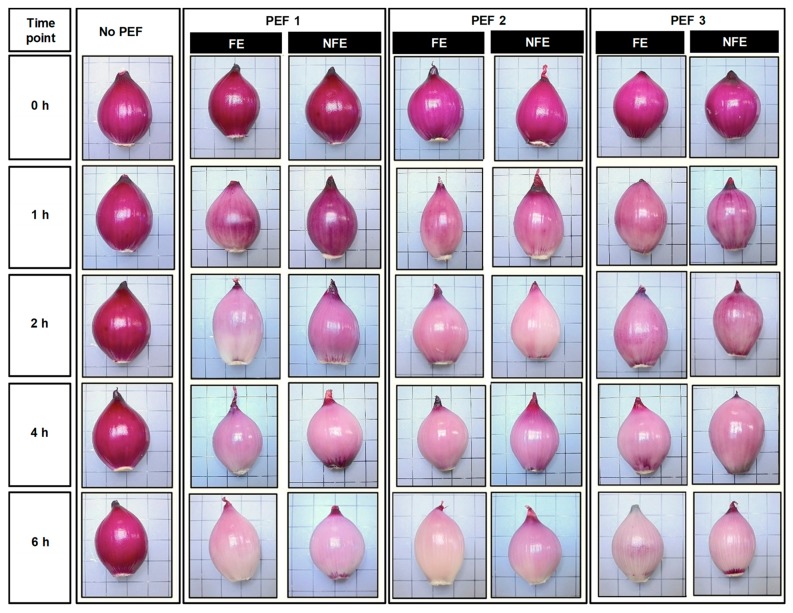
Representative photographs of untreated and PEF-treated onion bulbs over the course of 6 h after PEF application. The onion bulb was positioned with either the root end facing the electrode (FE) or the root end not facing the electrodes (NFE) at the time of PEF application, then suspended in the sodium phosphate buffer (0.01 M, pH 7) and removed at a predetermined time point to capture the changes in the natural purplish-red pigment distribution.

Immediately after PEF application (*t* = 0 h), the purplish red-colored pigments of onion found in the outermost fleshy scale (scale 1) of the bulb was not affected (Figure 3). A prominent reduction in the purplish-red pigments distributed in the outermost fleshy scale of the onion bulbs started to occur 1 h after PEF application. When comparing all PEF-treated onion bulbs at *t* = 1 h, it was observed that the PEF treatment efficacy on the onion bulb can be affected by the orientation of the onion at the time of PEF. In particular, onions treated at a low field strength and low energy (PEF 1, 0.6 kV/cm and 6 kJ/kg) in the NFE position experienced the least discoloration of purplish-red pigments in the outermost fleshy scales. At *t* = 6 h, all PEF-treated red onions generally lost most of their natural purplish-red pigments in the outermost fleshy scale, with some pigments still visible near to the foliar stem and disc stem. It is important to note that untreated onions experienced no changes in the purplish red-colored parts of the outermost fleshy scale over the course of 6 h steeping in the phosphate buffer. 

### 3.2. Changes in the Onion Epidermal Cells in Response to PEF

**Figure 4:**
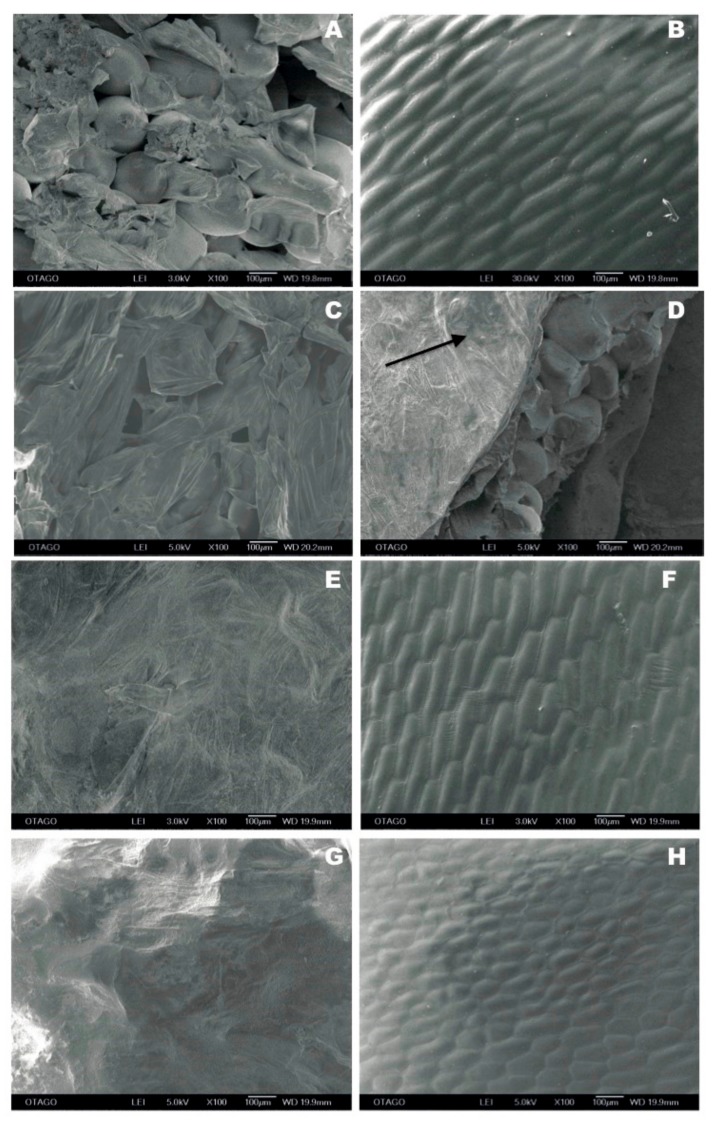
Cryogenic scanning electron micrographs of onion tissues observed (magnified view of 100×) at the abaxial (outer) and adaxial (inner) surfaces of the fleshy scales from untreated and PEF-treated onion bulbs. (**A**) The adaxial surface of scale 2 from an untreated onion. (**B**) The abaxial surface of scale 2 from an untreated onion. (**C**) The adaxial surface of scale 3 from an “NFE-PEF3” onion. (**D**) The adaxial surface of scale 3 from an untreated onion. (**E**) The adaxial surface of scale 2 from an “FE-PEF3” onion. (**F**) The abaxial surface of scale 2 from an “FE-PEF3” onion. (**G**) The adaxial surface of scale 3 from an “FE-PEF2” onion. (**H**) The abaxial surface of scale 3 from an “FE-PEF2” onion.

Representative cryo-SEM micrographs of untreated and PEF-treated onion tissues (Figure 4) showed that there was a considerable difference in the onion epidermal cells in response to PEF. Figure 4A shows the adaxial (inner) surface of fleshy scale 2 from an untreated onion bulb that was damaged during sample preparation (with the tunic layer removed), thus revealing intact and turgid underlying storage circular cells. Likewise, Figure 4B shows minimal damage on the abaxial (outer) surface of fleshy scale 2 from an untreated onion bulb, showing intact and turgid elongated epidermal cells beneath the cuticle layer. Abaxial epidermal cells had dimensions of approximately 250.44 ± 16.39 µm × 67.48 ± 3.80 µm (length × width) (*n* = 10); while, the relative isometric storage circular cell had a mean diameter of 204.20 ± 8.37 µm (*n* = 10). The effect of PEF applied at 1.2 kV/cm (PEF 3) with the NFE orientation on the adaxial surface of a PEF-treated onion was observed at the fleshy scale 3 (Figure 4C). The underlying storage circular cells had lost their turgidity and many cells were collapsed (Figure 4C), in comparison to the storage circular cells in the untreated onion scale 3 (Figure 4D), where the storage cells at the cut edge of the specimen were mostly intact and turgid. Some minor surface damage can be seen because of tunic layer removal, as shown in Figure 4D with an arrow. 

Severe cell damage caused by a “PEF 3” treatment with an onion orientation where its root end was facing the high voltage electrode (FE) can be seen on the adaxial surface of scale 2, as illustrated in Figure 4E. The observed flat surface was made up of dead cells that completely lost their cell wall integrity. On the abaxial surface of scale 2 obtained after a “FE-PEF3” treatment (Figure 4F), there was minimal surface damage, which was similar to the untreated scale 2 evaluated at the abaxial surface (Figure 4B). However, the severe damage on the storage circular cells (Figure 4E) caused a reduction in tissue thickness and density due to the loss of cytosol and cellular turgidity leading to wrinkle formation on the cuticle layer (Figure 4F; arrow).

Figure 4G,H shows the effect of a specific energy input of 60 kJ/kg (PEF 2) with the FE orientation on the adaxial and abaxial surfaces of a PEF-treated onion observed in fleshy scale 3. Figure 4G specifically shows that “FE-PEF2” caused a complete disruption of the adaxial cell layers and tissue structure similar to Figure 4E. In contrast, “FE-PEF2” caused minimal damage on the cuticle layer with the epidermal cells mostly intact and turgid (Figure 4H). It was also found that the epidermal cells became smaller and less elongated in the adaxial scales compared to the other abaxial scales in response to “FE-PEF2” treatment.

Overall, the results from cryo-SEM showed that PEF had minimal damage on the abaxial surface, whereas most damage was found on the adaxial surface of the fleshy scales. Abaxial epidermal onion cells retained their cell wall integrity following PEF treatment (Figure 4F,H); while the adaxial storage circular cells lost their cell wall integrity (Figure 4C,E,G). 

### 3.3. Effect of PEF on the Viability of Onion Cells

**Figure 5:**
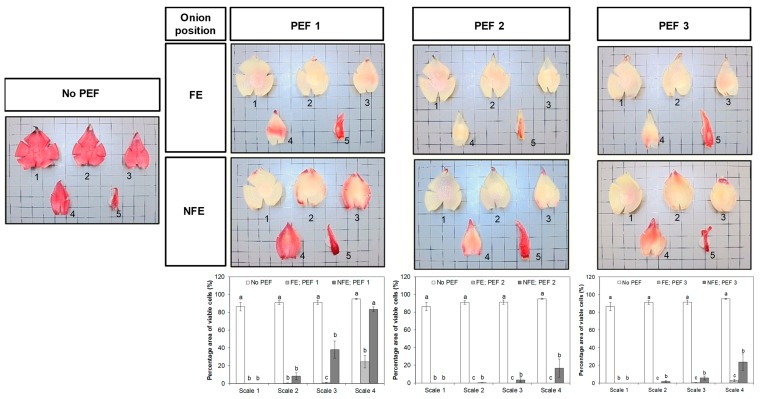
Representative photographs of onion scales from untreated (No PEF) and PEF-treated bulbs stained with tetrazolium salt and the percentage area fractions of viable cells (mean ± standard error, *n* = 3; bars with different lower-case letters indicate a significant difference (*p* < 0.05) between the onion orientations for each scale). Onion bulbs were positioned with either the root end facing the electrode (FE) or the root end not facing the electrodes (NFE) at the time of PEF application. Viable cells were stained red and dead cells were unstained. Number 1–5 indicates the fleshy scales and a terminal bud in an onion bulb, as designated in Figure 1.

All four fleshy scales and terminal bud detached from an untreated onion bulb were stained red with the tetrazolium dye, indicating more than 85% of the onion cells were found viable without a PEF pre-treatment (Figure 5). The effect of PEF treatment on the viability of onion cells intensified from the outermost to inner fleshy scales. The outmost fleshy scale (scale 1) of onion bulbs completely lost their cell viability as a result of PEF treatments, although some inner layers of onion scales and terminal buds had a better retention of cell viability. There was a clear effect of onion orientation at the time of PEF application on the cell viability of onions across the different scale layers. For example, the application of PEF, at an electric field strength of 0.6 kV/cm and a specific energy input of 6 kJ/kg (PEF 1), was more effective in reducing cell viability across the onion scales, particularly when the onion bulb was oriented with the root end positioned facing the high voltage electrode (FE).

With respect to onions placed in the “NFE” orientation, it was found that the third and fourth fleshy scales of onions pre-treated with PEF 1 retained a larger proportion of metabolically viable cells, with up to 83% of the cell areas still stained red, compared to PEF-treated samples placed in the “FE” orientation. Likewise, 17–24% of the total area of inner layers of onion scales (particularly scales 3 and 4) remained metabolically viable and were not affected by the “PEF 2” and “PEF 3” treatments when placed in the “NFE” position. Apart from onion orientation, increasing the intensity of the electric field strength from 0.6 to 1.2 kV/cm (PEF 1 vs. PEF 3) and specific energy from 6 to 60 kJ/kg (PEF 1 vs. PEF 2) caused more cell death. A higher proportion of onion cells in the inner layers of the fleshy scales detached from whole bulbs pre-treated at higher PEF intensities had lost their viability. 

### 3.4. Effect of PEF on the Concentration of Headspace Onion Volatiles

**Figure 6:**
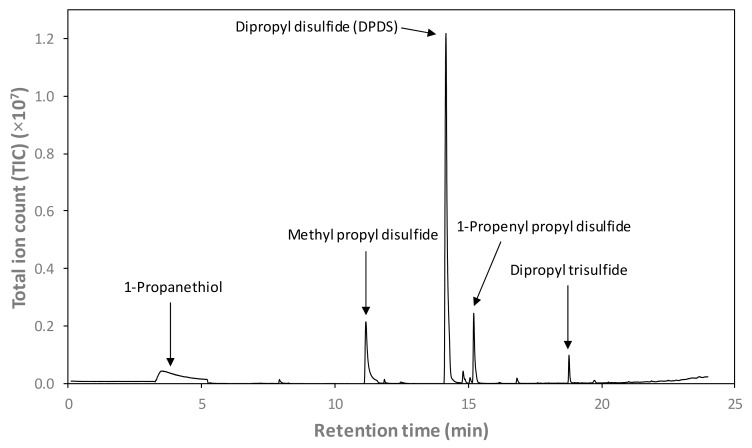
Total ion chromatogram of the headspace of the sodium phosphate buffer (0.01 M, pH 7) following 6 h of steeping with a PEF-treated onion bulb, obtained using solid-phase microextraction gas chromatography–mass spectrometry (SPME-GC-MS). Target peaks of interest are identified and labelled. Note that 1-propanethiol co-eluted with the broad air peak (≈3.2–5.2 min), quantified with an extracted ion (*m*/*z* 76).

Figure 6 depicts an example GC-MS total ion chromatogram (TIC) of the headspace of the sodium phosphate buffer following 6 h of steeping with a PEF-treated onion bulb. Five sulfur-containing volatile compounds of interest were identified: 1-propanethiol, 3 disulfides (methyl propyl disulfide, DPDS, and 1-propenl propyl disulfide), and dipropyl trisulfide. DPDS was the predominant volatile detected with the most abundant peak area in the headspace of the phosphate buffer following 6 h of steeping with onions. 

**Figure 7:**
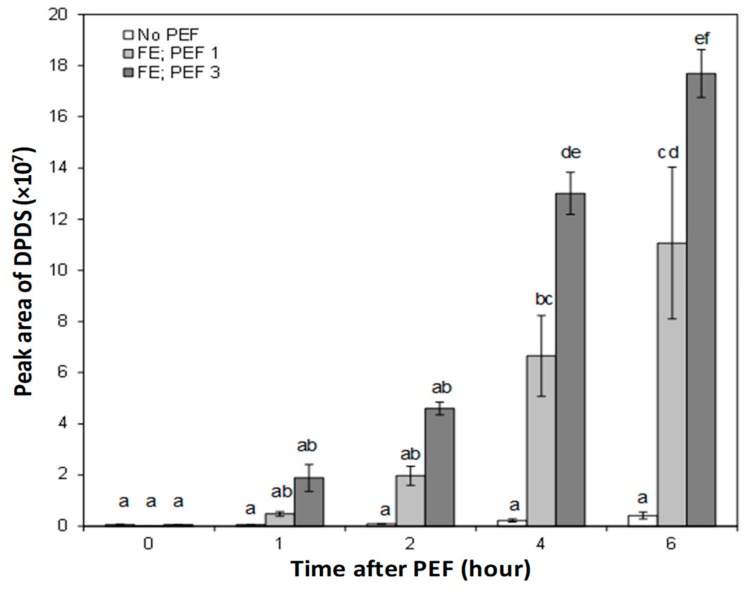
Extracted ion peak area of dipropyl disulfide (DPDS) (*m*/*z* 150) detected in the headspace of the sodium phosphate buffer over the course of 6 h of steeping with untreated and PEF-treated onion bulbs. Bars represent mean ± standard error (*n* = 3). Significant differences (*p* < 0.05) between the means are indicated with different letters.

The average peak areas of all detected onion volatile compounds in the phosphate buffer increased in response to PEF treatment, particularly when the onion bulb was oriented with the root end positioned facing the high voltage electrode at the time of PEF (FE). The headspace concentration of DPDS did not increase significantly during the first 2 h of steeping for both untreated and PEF-treated onion bulbs. Following 4 and 6 h of steeping with onions pre-treated with PEF, the amount of DPDS increased significantly in the phosphate buffers (Figure 7). The greatest increase in DPDS (up to a 59-fold more than an untreated onion) was found in the headspace of the phosphate buffer steeped for 6 h with an onion treated with an electric field strength of 1.2 kV/cm (PEF 3) and positioned facing the high voltage electrode (FE) at the time of PEF application.

Without a PEF pre-treatment on the onion, it was demonstrated that the peak area of DPDS in the headspace of the phosphate buffer over the course of 6 h of steeping was almost at the same level and never exceeded more than 0.5 × 10^7^ (Figure 7). In fact, changes in the DPDS derived from untreated onions as a function of steeping duration were not statistically significant from one time point to another (*p* > 0.05). 

The kinetic changes in the volatile compounds isolated in the headspace of the phosphate buffer during steeping with untreated onions were the slowest, with a kinetic rate constant (*k*) estimated at (0.06 ± 0.01) × 10^7^ peak area per hour (Table 2). In contrast, it was found that DPDS in the headspace of the phosphate buffer for PEF-treated onions increased linearly with the steeping duration (Figure 7). Overall, the results showed that the rate of DPDS increase (*k*) was accelerated, by at least 32- and 52-fold, in the headspace of the phosphate buffer steeped with onions PEF-treated at 0.6 (FE-PEF1) and 1.2 kV/cm (FE-PEF3), respectively, compared to untreated onion bulbs (Table 2). 

## 4. Discussion

PEF processing can result in different levels of structural damage in heterogeneous material such as plant tissue. Fincan and Dejmek [9] demonstrated that the distribution of permeabilized cells was non-homogenous across a one-cell-thick layer of abaxial onion epidermal tissue if treated with PEF at an electric field strength below the critical value (<0.33 kV/cm). While it has been well evidenced that irreversible cell damage of onion epidermal tissues usually occurs at an electric field strength greater than 0.33 kV/cm [9,15], these major structural changes may not be the case for all onion cells across the fleshy scale layers when PEF was used to treat a whole, intact bulb. In this study, validation of PEF efficiency to achieve irreversible and uniform disruption of cellular tissues was achieved using a combination of simple, yet reliable measurements, namely cell viability staining using tetrazolium salt and microscopic evaluation using cryo-SEM. Based on the findings, to achieve uniform and high levels of cellular damage and complete structural changes, PEF conditions should be designed to employ a higher electric field strength (1.2 kV/cm) at fewer pulses, to result in low energy input (6 kJ/kg), and using an onion orientation with the root end of the bulb facing to the high voltage electrode.

The purplish red-colored parts in the dried coats and outer parts of the fleshy scale-leaves of the bulbs of the red onion are attributed to the anthocyanin pigments such as cyanidin-3-glucoside, cyanidin-3-laminaribioside, cyanidin-3-malonyl-glucoside, and cyanidin-3-malonyl-laminaribioside [21]. In the present study, changes in the onion anthocyanins due to PEF were not measured directly; however, the results clearly demonstrated that PEF-treated onions had reduced the purplish-red colored pigments distributed in the outermost fleshy scale (Figure 3), indicating that PEF led to a loss of anthocyanins, possibly due to leaching or degradation. Changes in the color and stability of anthocyanins have been known to be affected by pH, temperature, light, oxygen, metal ions, and chemical association with other compounds [22]. However, the actual mechanism to explain whether PEF has direct involvement in causing the chemical degradation or instability of anthocyanins in onion cellular tissues is not the focus of the present study.

Anthocyanins are water-soluble pigments localized in the vacuole of the plant cell and protected by a membrane known as tonoplast. Asavasanti and others [10] have demonstrated that PEF treatment on onion tissues at a low field strength (0.07 kV/cm) can cause initial membrane breakdown at the cell membrane, while an electric field strength beyond 0.2 kV/cm could also lead to tonoplast membrane breakdown. Therefore, a possible explanation for the loss of purplish-red pigments in the onion cells could be that the PEF conditions under investigation were able to permeabilize the tonoplast of the onion cell. As a result, leaching of anthocyanins from the vacuole to the intra- or extra-cellular spaces would take place while inducing massive mixing of the cell contents at the vacuole, the cytoplasm, and the cellular spaces that completely reduced anthocyanins to their colorless form following PEF. 

Further evidence from the cryo-SEM micrographs revealed that storage circular cells at the adaxial surface experienced a considerable loss of cell integrity and turgidity (Figure 4E). Intact cell membranes were no longer distinguishable from the onion specimen and the effect of PEF under the investigated conditions on onion microstructure was even more severe than onion tissues processed with high pressure technology [23,24]. Therefore, it can be suggested that the cell membrane and the tonoplast of onion cells within the bulb showed lesser resistance to PEF treatments, and hence, exhibited a rather significant PEF-induced cell disruption. 

Onion cells, depending on their cell size, cell type, and distribution of cells, have been found to exhibit different abilities to resist cell damage due to PEF [9,15]. Therefore, cell viability staining tests have been successfully applied in other studies to visualize the uniform distribution of the PEF effect across all the onion scales within the bulb [9,15]. An important finding from the cell viability test in this study was that the onion orientation with respect to the high voltage electrode at the time of PEF had an important role in causing uniform cell death (Figure 5). This was most possibly due to the fact that cells that were parallel to the applied electric field (onion bulb at “FE” position) were more prone to cell membrane breakdown [25]. Moreover, during the PEF treatment, onion bulbs with the root end facing the high voltage electrode may create less electrical resistance toward the incoming high voltage pulses and this onion orientation may confine the flow of electric voltage in one direction: i.e., entering from the root end, through the individual fleshy scales, and exiting at the foliar stem. As a result, onion cells within the bulb in the “FE” orientation had all areas across the scales that were found to be uniformly non-viable (Figure 5). In contrast, during the initiation of electric pulses, onion bulbs with the root end not facing the high voltage electrode (“NFE” orientation) may experience electrical resistance from the cuticle layer at each onion scale and the flow of electric charge can be easily dispersed in different directions within the onion bulb. It was not unexpected that terminal buds were unaffected by PEF, under any of the PEF conditions investigated, because the terminal bud has different cell types and arrangements compared to onion cells in the fleshy scale [26].

From the results of the present study, it is clear that PEF can causes major uniform cell damage and major structural changes across all onion cells within the bulb, provided the correct PEF conditions and sample orientation are chosen. Therefore, structural damage due to PEF may initiate enzymatic reactions, including those catalyzed by alliinase, within the whole and intact onion bulb. The breakdown of cell membranes and tonoplasts due to PEF, leading to the loss of cell integrity and absence of cell compartmentalization [10], most probably promoted the release of sulfur-containing precursors (ACSOs) and the alliinase enzyme from their localization in onion cells. As a result, this would facilitate the mixing of these compounds, resulting in the formation of the characteristic onion odor and flavor, and hence higher concentrations of sulfur-containing volatile compounds are expected to be detected.

Onions have a complex volatile profile in which different classes of compounds can be detected either in the headspace or in the onions. In this study, PEF-treated onion bulbs were steeped in a phosphate buffer for a predefined duration following treatment in order to measure compounds that were generated within the onion bulb after PEF and released from the onion cells to the extracellular space. This method was selected to evaluate the changes occurring as a result of cellular disruption in the whole, intact onion bulb without the need for further destructive sample preparation, which could have generated more compounds as an artefact. This method also allowed for the accumulation of compounds of interest from the same onion bulb over the 6 h period. Remarkable increases in DPDS were detected in the headspace of the phosphate buffer over time due to its high concentration, high volatility, and affinity for the SPME adsorbent phase. DPDS and the other two disulfides detected in the headspace are products of disproportionation of the unstable thiosulfonates, which are subsequent products from the condensation of sulfenic acid originating from the alliinase-catalyzed enzymatic reaction [27]. This indicates that most of the volatiles detected in the headspace of phosphate buffers steeped with PEF-treated onion bulbs were generated by the alliinase-catalyzed reaction pathway. 

Intact onion cells have no characteristic flavor generated until cellular disruption occurs. However, a small amount of DPDS detected in the headspace of phosphate buffers from an untreated onion bulb could be due to differences in turgor pressure occurring in some onion cells during a prolonged duration of steeping, thus leading to minor cell damage. Such cell damage can be considered negligible since the formation and release of DPDS did not take place rapidly as compared to after the PEF treatment. The present result showed that the formation of characteristic onion sulfur-containing volatile compounds in a whole, intact onion bulb following a PEF treatment involved a time-dependent enzymatic-catalyzed reaction (Table 2). After the rupture of the onion cell structure in response to PEF treatment, it appears that it will take some time after PEF for the enzymatic reaction to occur within the bulb and for the generated volatiles to be released to the extracellular space. It will be of future interest to determine whether 6 h post PEF treatment was sufficient for no further increase in DPDS concentration to take place. 

## 5. Conclusions

Results from the present work showed that PEF treatment on whole, intact red onion bulbs, if applied under appropriate conditions, is capable of causing severe microstructural damage. The orientation of the onion bulb with respect to the PEF electrode, which is associated with the different positions of onion cells within the bulb exposed to the applied electric field at the time of PEF, was found to have a major influence on the effectiveness of the applied PEF condition to cause severe, yet consistent, structural changes. Meanwhile, PEF-treated onion bulbs were found to release an increased concentration of DPDS as a function of time after the PEF application. It is likely that structural changes in onion tissue structure induced by PEF facilitated the enzyme-catalyzed reactions of alliinase leading to the generation of distinctive sulfur-containing volatiles such as DPDS. This study also suggests that PEF can be used as a tool to cause cellular disruption within a whole, intact solid plant tissue to initiate desirable enzymatic reactions leading to the generation of potentially useful metabolites before transforming them into edible food.

## Figures and Tables

**Table 1:** Parameters of three different Pulsed electric fields (PEF) treatments.

PEF Treatment	Electric Field Strength (kV/cm)	Pulse Width (µs)	Pulse Frequency (Hz)	Pulse Number	Specific Energy Input (kJ/kg)
PEF1	0.6	20	50	540	6
PEF2	0.6	20	50	5400	60
PEF3	1.2	20	50	120	6

**Table 2:** Estimated kinetic parameters based on a zero-order kinetic model describing increases in DPDS, detected in the headspace of the sodium phosphate buffer (0.01 M, pH 7), over the course of 6 h of steeping with untreated and PEF-treated onion bulbs.

PEF Treatment	*k* (×10^7^ Peak Area Per Hour)	Corrected R^2^
No PEF	0.06 ± 0.01	0.6805
FE; PEF 1	1.94 ± 0.29	0.7589
FE; PEF 3	3.12 ± 0.15	0.9671

Data presented as estimated kinetic parameter ± asymptotic standard error of estimated parameter at 95% confidence intervals using SAS 9.4 based on a zero-order linear regression analysis (Equation (1)).
